# Paradigm Shift in Drug Re-purposing From Phenalenone to Phenaleno-Furanone to Combat Multi-Drug Resistant *Salmonella enterica* Serovar Typhi

**DOI:** 10.3389/fcimb.2018.00402

**Published:** 2018-11-14

**Authors:** Shama Mujawar, Derek Gatherer, Chandrajit Lahiri

**Affiliations:** ^1^Department of Biological Sciences, Sunway University, Bandar Sunway, Malaysia; ^2^Department of Biomedical and Life Sciences, Lancaster University, Lancaster, United Kingdom

**Keywords:** drug repurposing, salmonellosis, multidrug resistance, chaperones, SicA, DnaK

## Abstract

Over recent years, typhoid fever has gained increasing attention with several cases reporting treatment failure due to multidrug resistant (MDR) strains of *Salmonella enterica* serovar Typhi. While new drug development strategies are being devised to combat the threat posed by these MDR pathogens, drug repurposing or repositioning has become a good alternative. The latter is considered mainly due to its capacity for saving sufficient time and effort for pre-clinical and optimization studies. Owing to the possibility of an unsuccessful repositioning, due to the mismatch in the optimization of the drug ligand for the changed biochemical properties of “old” and “new” targets, we have chosen a “targeted” approach of adopting a combined chemical moiety-based drug repurposing. Using small molecules selected from a combination of earlier approved drugs having phenalenone and furanone moieties, we have computationally delineated a step-wise approach to drug design against MDR *Salmonella*. We utilized our network analysis-based pre-identified, essential chaperone protein, SicA, which regulates the folding and quality of several secretory proteins including the Hsp70 chaperone, SigE. To this end, another crucial chaperone protein, Hsp70 DnaK, was also considered due to its importance for pathogen survival under the stress conditions typically encountered during antibiotic therapies. These were docked with the 19 marketed anti-typhoid drugs along with two phenalenone-furanone derivatives, 15 non-related drugs which showed 70% similarity to phenalenone and furanone derivatives and other analogous small molecules. Furthermore, molecular dynamics simulation studies were performed to check the stability of the protein-drug complexes. Our results showed the best binding interaction and stability, under the parameters of a virtual human body environment, with XR770, a phenaleno-furanone moiety based derivative. We therefore propose XR770, for repurposing for therapeutic intervention against emerging and significant drug resistance conferred by pathogenic *Salmonella* strains.

## Introduction

Bacterial infections have been a life threatening problem to the human population throughout the existence of our species. In addition, with the advent of antimicrobial resistance (AMR), this has become an ever-increasing concern, contributing nearly one fifth of total global deaths every year. With a worldwide prevalence of probably five hundred million cases and hundreds of thousands of deaths every year, human salmonellosis or typhoid fever, has become a major cause for concern with the emergence of multidrug-resistance (MDR) serovars and strains of *Salmonella enterica* (Zaidi et al., 2008). The severity of the disease, depends on host factors and the individual emerged MDR serotype of *Salmonella* (Neckers and Tatu, 2008). Thus, it has become imperative to replace conventional anti-typhoid treatment strategies with new ones more suitable for drug-resistant pathogens. However, designing novel effective drugs against the most important virulence protein targets, choosing from the whole genome of *Salmonella* sp., is a highly challenging task.

To encounter the aforementioned threats and challenges of MDR, new strategies like drug repurposing have surfaced as alternative approaches to novel therapeutics (Rangel-Vega et al., 2015). This include computational drug repurposing strategies based on transcriptional signatures, networks, ligands, target structures using chemogenomics, machine learning, and molecular docking techniques (March-Vila et al., 2017). Nevertheless, drug repurposing approach may have some limitations on its successful outcome (Aubé, 2012). For example, the drug might act at the same target but with different outcomes that depend on the new active site of biological action of the repurposed drug (Aubé, 2012). Again, different structure-activity relationship might be possible for old and new drug with different biochemical targets, leading to an obsolete solution to be replaced by further repurposed drugs (Aubé, 2012). However, a screening of drugs identified for repurposing has proven to be useful when used together in combination thereby making it the most competing strategy to adapt the current pharmacopeia for new uses (Aubé, 2012).

To repurpose drug(s) through a network based approach, the technique of computationally analyzing the protein interaction networks for *Salmonella* could be employed (Pan et al., 2015). This pathogen is found to encode a Type III Secretion System (T3SS) within a pathogenicity island that is essential for virulence (Tucker and Galán, 2000). All T3SSs require the function of a family of low-molecular-weight proteins that aid the secretion process by acting as secretion factors (Tucker and Galán, 2000). One such secretion associated protein is the chaperone SicA, identified as a secretion factor from the network based approach of the protein interactome analysis of *Salmonella* pathogenicity islands (SPI) and two component signal transduction systems (TCS) (Lahiri et al., 2014). In summary, chaperones aid in folding, packaging, and secretion of synthesized proteins besides inhibiting aggregation due to stress factors like heat shock, thereby striking a balance between refolding and proteolytic degradation (Liberek et al., 2008). The potential of exploiting pathogenic chaperones as drug targets has also been reported (Neckers and Tatu, 2008). Notably, chaperones that support folding of newly synthesized proteins, namely Hsp60, Hsp70, and Hsp90, have distinct mechanisms of action (Fink, 1999). Most of these heat shock proteins (HSP) are constitutively synthesized but are further induced in response to stress conditions, including antibiotic therapies (Zügel and Kaufmann, 1999). In fact, DnaK, the bacterial homolog of human Hsp70, has been found to play an important role in pathogen survival (Chiappori et al., 2015). Moreover, previous work has shown that the chaperone SicA, is auto-regulated and required for the transcription of *sigE* (Darwin and Miller, 2001). SigE is another Hsp70 molecular chaperone protein required for stabilization of SopB/SigD secretory proteins through prevention of premature association of effectors and their degradation prior to secretion (Darwin et al., 2001). All of these proteins could, therefore, form the new targets for the newly proposed drug for repurposing. Targeting such HSPs may, therefore, help to combat pathogen virulence by reducing its capacity to respond to other treatments (Neckers and Tatu, 2008). Such treatments have been linked to the therapy of inflammatory diseases and cancer which is possible due to the molecular chaperone and antigen-binding properties of the HSPs (Calderwood et al., 2012).

To repurpose drugs through a ligand based approach, a combination of the chemical moieties of individually approved and marketed drugs could be used. Typically, drugs used mostly for treating infectious diseases caused by gram positive pathogens contain phenalenone moieties (March-Vila et al., 2017). Again, furanone moiety-based drugs have gained attention for treating other infectious (Chrystal et al., 2007). To address the pressing need for better treatment alternatives for MDR *Salmonella*, here we have used a combined phenaleno-furanone moiety-based ligand XR770, which was previously shown to be more preferable in performing as dual TCS inhibitor against the catalytic domain of the histidine kinase BaeS and the dimerization domain of the response regulator BaeR (Sivakumar et al., 2013). In summary, using molecular dynamics simulation, we have screened current typhoid drugs, non-typhoidal drugs and Hsp70 modulators to propose XR770 as a drug candidate against plausible chaperone protein targets, namely SicA, DnaK, and SigE compared to our reference TCS protein pairs, SsrA/SsrB and OmpR/EnvZ of the T3SS. We anticipate that targeting such molecular chaperones might help in the rational development of effective drugs for salmonellosis and thus, future control of the pathogenic bacterial growth, in an era of rapidly increasing antibiotic resistance. Furthermore, our combined-moiety ligand-based molecular docking approach via indispensable targets, will likely provide new opportunities of drug repurposing for MDR *Salmonella*.

## Materials and methods

### Selection of ligands

Ligand screening was performed with the aim of finding existing drugs for treating systemic infections like typhoid (Kaur et al., 2011). The molecular structures of the 19 typhoid-related drugs were obtained from Drug Bank (Wishart et al., 2017), namely Amoxicillin, Azithromycin, Ceftriaxone, Chloramphenicol, Ciprofloxacin, Levofloxacin, Ofloxacin, Sulfamethoxazole, Trimethoprim, Rolitetracycline, Auranofin, Imiquimod, Alverine, Lymecycline, Digitoxin, Rimonabant, Isosorbide Mononitrate, Acenocoumarol, Doxycycline. Structures of phenalenone-furanone derivatives XR770 and XR587 were produced using MarvinSketch (Csizmadia, 1999). Furthermore, we constructed a control set of 15 other drugs, not known to cure typhoid, but already used to treat infections caused mostly by gram positive bacteria like *Staphylococcus aureus, Streptococcus pneumonia* etc. (Supplementary Table 2). The structures of these drugs, containing moieties of either phenalenone or furanone, were obtained from DrugBank *viz*. Moxifloxacin, Grepafloxacin, Lomefloxacin, Gatifloxacin, Sparfloxacin, Temafloxacin, Nemonoxacin, Besifloxacin, Finafloxacin, Cadazolid, Nadifloxacin, Sitafloxacin, Clinafloxacin, Pilocarpine, Matairesinol. The non-typhoidal drugs were found to possess 70% similarity to Phenaleno-furanone derivatives such as XR770 and XR587 (Sivakumar et al., 2013). Also, Hsp70 modulators (analogous to Hsp90 inhibitors) were also used in this study that are known to have significant effects on chaperone function (Patury et al., 2009).

### Selection of targets

The secretion associated chaperone protein, SicA, was selected on the basis of previous work (Lahiri et al., 2014) on the interactome analysis of SPI and TCS. Another chaperone protein, DnaK, of the Hsp70 class, was identified from the top-most centrality ranking protein list of the whole genome protein interaction network analysis of *Salmonella* Typhimurium strain LT2 (Mujawar & Lahiri, unpublished data). For a comparison with these identified targets, a similar Hsp70 chaperone protein, SigE, required for the stabilization of secretory proteins, was selected. These chaperone proteins were compared with the TCS protein pairs of SsrA/B and EnvZ/OmpR as controls for the binding efficacy of the ligand XR770 previously reported to be effective for the TCS pair BaeS/R of *Salmonella* (Sivakumar et al., 2013). As both the serovars of *Salmonella*, namely Typhimurium and Typhi share almost similar genes/proteins, the aforementioned proteins of Typhimurium have been utilized in the context of Typhi as well. The sequences of target chaperone proteins, SicA, DnaK, SigE, and the TCS proteins SsrA, SsrB, OmpR and EnvZ were collected from UniprotKB database with the accession IDs are P69066, Q56073, O30917, Q8ZPP5, O54305, P0AA19, and P0AA20 for *Salmonella* Typhimurium and P69065, Q8Z9R1, Q8Z7R2, Q8Z6K9, Q8XFU4, P08982, and P41406 for *Salmonella* Typhi, respectively.

### Target structure modeling

As the selected proteins above did not have any solved X-ray crystallographic or NMR 3D structures in Protein Data Bank (PDB), we have generated homology models and validated their structure to pursue further docking studies. We have used Phyre2 (Kelley et al., 2015), SWISS MODEL (Schwede et al., 2003) and I-TASSER (Zhang, 2008) and VERIFY 3D (Bowie et al., 1991) protein modeling servers to generate the structures and evaluated them through Ramachandran plot, Q-mean score, and Z-score listed in SAVES server. Consensus studies of different models generated by above mentioned servers was carried out to identify the best structure. Table 1 represents the details of protein structures that were used for further docking studies.

**Table 1:** Comparison of the 3D structures generated for SicA, DnaK, and SigE using different protein modeling servers.

**Protein**	**Server**	**Q-mean**	**Z-score**	**Favored region(%)**	**Disallowed region(%)**	**SASA (A^2^)**
SicA	**PHYRE2**	**0.765**	−**0.23**	**95.5**	**0.0**	**101**
	SWISSMODEL	0.727	−0.55	94.7	0.0	97
	I-TASSER	0.481	−2.84	87.0	0.0	103
	VERIFY 3D	0.761	−0.26	93.2	0.0	91.6
DnaK	PHYRE2	0.686	−0.86	89.7	0.2	106
	SWISSMODEL	0.621	−0.81	81.0	0.5	105
	**I-TASSER**	**0.689**	−**0.80**	**90.5**	**0.1**	**106**
	VERIFY 3D	0.692	**–**0.67	89.4	0.1	106
SigE	PHYRE2	0.712	−0.40	89.2	0.2	91
	SWISSMODEL	0.741	−0.26	80.1	0.2	96
	**I-TASSER**	**0.622**	–**0.73**	**91.6**	**0.0**	**99.1**
	VERIFY 3D	0.644	**–**0.23	89.2	0.2	91.3
SsrA	PHYRE2	0.721	−0.43	90.1	0.1	92.3
	SWISSMODEL	0.702	−0.55	89.0	0.1	85
	**I-TASSER**	**0.753**	–**0.82**	**92.2**	**0.0**	**89.1**
	VERIFY 3D	0.719	−0.56	90.2	0.0	89.2
SsrB	PHYRE2	0.681	−0.81	92.2	0.2	101
	**SWISSMODEL**	**0.723**	–**0.80**	**90.2**	**0.1**	**89.2**
	I-TASSER	0.695	−0.83	90.0	0.2	88.1
	VERIFY 3D	0.69	−0.81	90.2	0.1	88.2
OmpR	PHYRE2	0.721	−0.29	92.5	0.0	96.1
	**SWISSMODEL**	**0.786**	−**0.43**	**96.2**	**0.0**	**104.0**
	I-TASSER	0.681	**–**0.43	94.3	0.0	93.1
	VERIFY 3D	0.732	**–**0.43	94.9	0.0	101
EnvZ	**PHYRE2**	**0.771**	−**0.36**	**94.6**	**0.0**	**101**
	SWISSMODEL	0.762	−0.33	94.4	0.0	100
	I-TASSER	0.769	**–**0.33	93.2	0.0	98.3
	VERIFY 3D	0.786	**–**0.34	94.4	0.0	98.0

*Bold values indicates best representative*.

### Binding site identification for docking

To have an understanding of the binding activity, active sites or binding pockets of the selected proteins were determined by using CASTp server (Computer Atlas of Surface Topology of protein) (Dundas et al., 2006). AutoDock v4.2 was used to generate grid box and map files for docking (Morris et al., 2009). The generated grid coordinates *viz*. X, Y, and Z were stored in a grid parameter file (GPF). The atoms such as Hydrogen (H), chlorine (Cl), bromine (Br), sulfur (S), phosphorus (P), and fluorine (F) were added, as appropriate, to set the map types to generate the grid box that covers the active site. The span of the active sites in the 3D structures of SicA, DnaK, SigE, SsrA, SsrB, OmpR, and EnvZ on X, Y, & Z coordinates were 40, 7, and 17 Å, respectively. The grid dimensions for the receptor for docking were taken as 60, 60, and 60 Å, respectively, to ensure that the search spaces are large enough for the ligands to rotate in. The execution of GPF files using MGLTools v1.5.7 was used to generate the map files of the atoms mentioned above to be used for docking. Upon GPF execution, grid log files (GLG) were generated which consisted of all the atomic map files to be used as the input parameter for docking calculated by the program AUTOGRID (Morris et al., 2009).

### Ligand screening against salmonellosis

**Figure 1:**
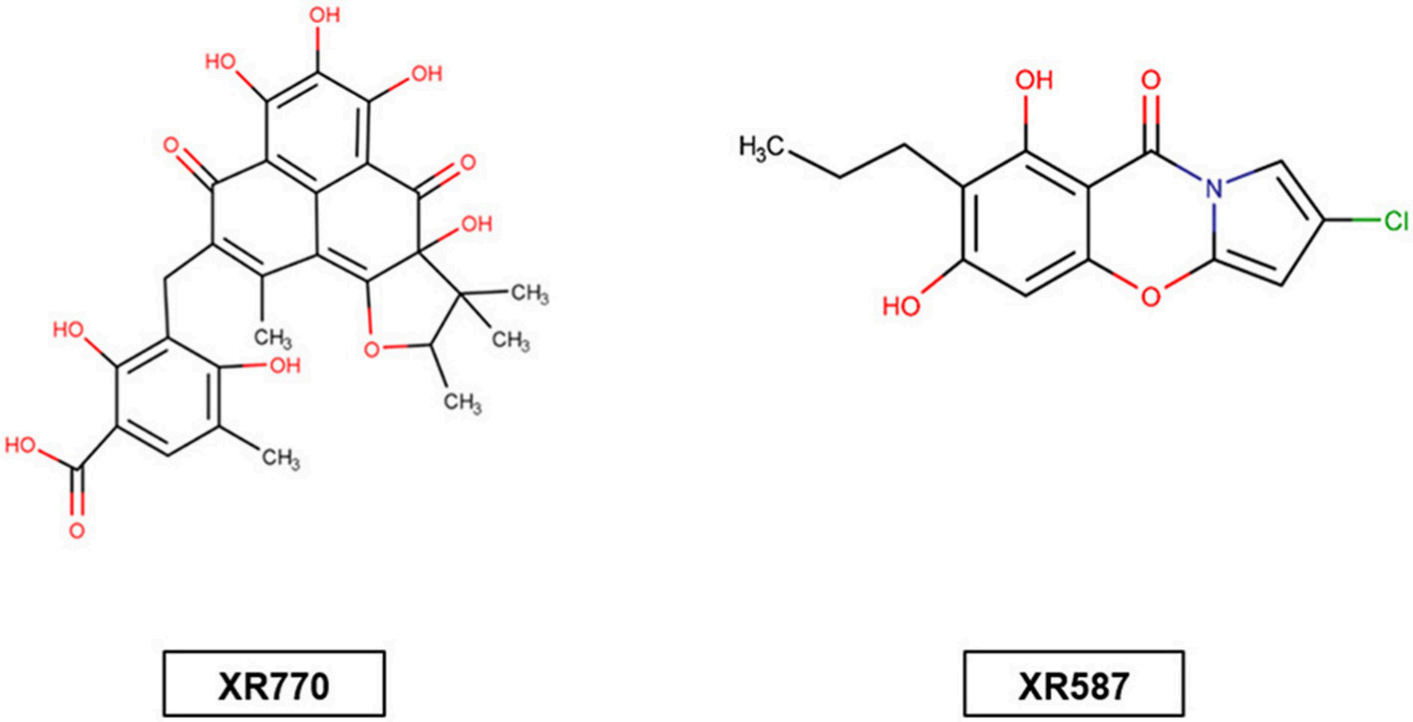
Structure of signal transduction inhibitors XR770 (a phenalenone derivative) produced by Penicillium cf. herquei 20421 and XR587 (streptopyrrole) fermentation product of actinomycete strain.

**Figure 2:**
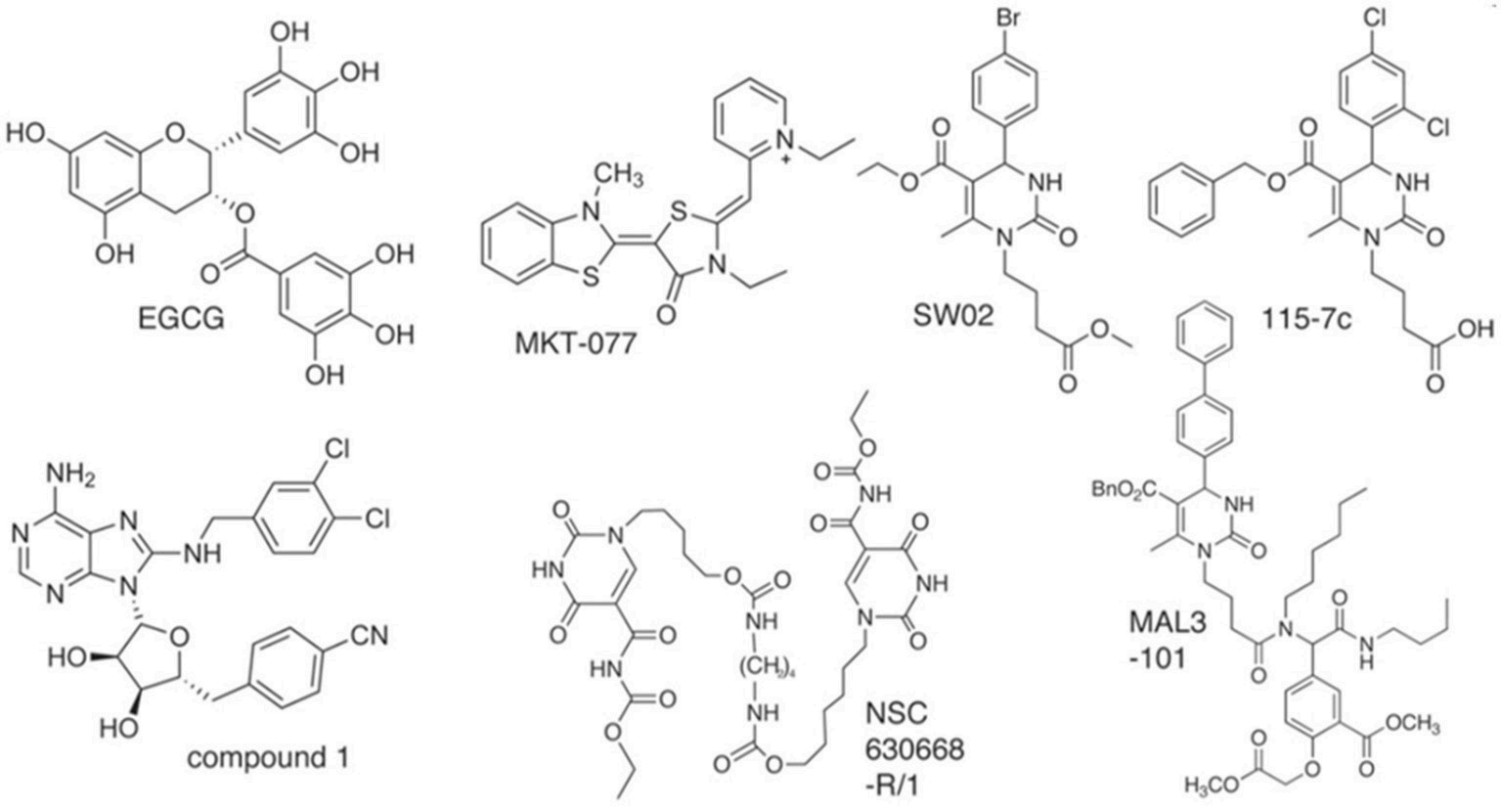
Structures of Hsp70 modulators (analogous to Hsp90 inhibitors) with selective chemical scaffolds that target the varied functions of chaperones.

The aforementioned existing drugs, along with the hypothesized **signal** transduction inhibitors (Chrystal et al., 2007), were used for chaperone protein inhibition as shown in Figures 1, 2. XR770 and XR587 were further analyzed on the basis of Lipinski Rule of five. ADME and Toxicity testing were also done using Schrodinger Software (Friesner et al., 2006).

### Lipinski's rule prediction of selected drugs

To evaluate drug likeness and determine if a chemical compound with a certain pharmacological or biological activity has properties that would make it a likely orally active drug in humans, we have carried out the Lipinski's rule prediction method for the selection of drugs. Comparison of the marketed drugs reflected some deviation to the above rule but are still used for oral administration with the given inhibitory concentration (Tables 2, 3).

**Table 2:** Physicochemical properties of typhoid related drugs and phenaleno-furanone derivatives.

**Drugs**	**Compound_ID**	**Molecular weight g/mol**	**XLOGP3**	**HBond donor**	**HBond acceptor**	**Inhibitory conc. (mol/ Kg)**
Acenocoumarol	DB01418	353.33	2.5	1	6	2.7869
Alverine	DB01616	281.443	5.3	0	1	2.6539
Amoxicillin	DB01060	365.404	−2.0	4	7	1.7036
Anacardic Acid	167551	348.527	9.5	2	3	NA
Auranofin	DB00995	678.483	NA	0	10	3.1438
Azithromycin	DB00207	748.996	4.0	5	14	2.5423
Ceftriaxone	DB01212	554.571	−1.3	4	13	2.1681
Chloramphenicol	DB00446	323.126	1.1	3	5	2.2247
Ulexone C	14583602	420.461	4.3	2	6	NA
CID_21591963	21591963	438.476	3.4	3	7	NA
Ulexin C	5323553	418.445	4.4	2	6	NA
Osajin	95168	404.462	5.9	2	5	NA
Ciprofloxacin	DB00537	331.347	−1.1	2	7	NA
Digitoxin	DB01396	764.95	2.3	5	13	4.4764
Doxycycline	DB00254	444.44	−0.7	6	9	2.3159
Ergonovine	DB01253	325.412	1.8	3	3	3.3967
Imiquimod	DB00724	240.31	2.6	1	3	2.5683
Isosorbide_Mononitrate	DB01020	191.139	−0.4	1	6	2.0753
Levofloxacin	DB01137	361.373	−0.4	1	8	NA
Lymecycline	DB00256	602.641	−4.4	9	13	2.5422
Ofloxacin	DB01165	361.373	−0.4	1	8	2.1639
Rimonabant	DB06155	463.787	6.5	1	3	2.5418
Rolitetracycline	DB01301	527.574	−0.9	6	10	2.7094
Sulfamethoxazole	DB01015	253.276	0.9	2	6	1.6422
Trimethoprim	DB00440	290.323	0.9	2	7	1.7701
XR587	NA	281.611	−0.5	2	7	NA
**XR770**	**NA**	**512.301**	−**0.6**	**2**	**8**	**NA**

*Bold values indicates best representative*.

**Table 3:** Physicochemical properties of 70% similar phenalenone-furanone derivatives.

**Drugs**	**Compound_ID**	**Molecular weight g/mol**	**XLOGP3**	**HBond donor**	**HBond acceptor**	**Inhibitory Conc. (mol/ kg)**
Moxifloxacin	DB00218	401.438	0.6	2	8	2.3267
Grepafloxacin	DB00365	359.401	−0.2	2	7	2.0923
Lomefloxacin	DB00978	351.354	−0.8	2	8	1.9971
Gatifloxacin	DB01044	375.4	−0.7	2	8	2.3029
Sparfloxacin	DB01208	392.407	0.1	3	9	1.9265
Temafloxacin	DB01405	417.388	0.6	2	9	2.0973
Nemonoxacin	DB06600	371.437	0.3	2	7	NA
Besifloxacin	DB06771	393.84	0.7	2	6	2.3263
Finafloxacin	DB09047	398.394	−0.5	2	8	NA
Cadazolid	DB11847	585.561	1.4	3	10	NA
Nadifloxacin	DB12447	360.385	0.8	2	6	NA
Sitafloxacin	DB13261	873.68	0.2	2	6	NA
Clinafloxacin	DB14025	365.79	0.3	2	6	NA
Pilocarpine^*^	DB01085	208.256	1.1	0	3	2.6826
Matairesinol^*^	DB04200	358.385	2.7	2	5	2.4961

The asterisk

(*) *indicate the furanone derivatives*.

### Protein-ligand docking

To perform the docking process, a genetic algorithm is used to furnish the docking conformations, binding energies, interactions etc. For the docking of ligands into intended protein binding pockets (Kitchen et al., 2004) and to approximate the binding affinities of the ligands, the molecular docking program AutoDock Vina was used (Trott and Olson, 2010). Docking studies were performed on SicA, DnaK, SigE, SsrA, SsrB, OmpR, and EnvZ against 21 typhoid related drugs (Table 4 and Supplementary Figure 2), 15 non-typhoidal drugs (Table 5 and Supplementary Figure 3), and 7 Hsp70 modulators (Table 6 and Figure 2). The protein and the ligand files were changed into the PDBQT format containing the protein atom coordinates, partial charges and deliverance parameters. Auto Grid boxes were predetermined around the active site of the protein based on the Lamarckian Genetic Algorithm (LGA) and the obtained dock scores were reported in kcal/mol. The utilized docking protocol comprised 35 autonomous iterations per ligand. The docked log files (DLG) calculated by the program AutoDock consisted of detailed information about binding energy, hydrogen bonds, interacting residues etc. which were further used for the docking analysis (Sousa et al., 2006).

**Table 4:** Interaction pattern of SicA, DnaK and SigE with the corresponding interacting residues and Binding energy against typhoid related drugs.

**Drugs**	**SASA**	**Interacting residues**			**Binding energy**			**H bonds**		
	**(A^2^)**	**SicA**	**DnaK**	**SigE**	**SicA**	**DnaK**	**SigE**	**SicA**	**DnaK**	**SigE**
Amoxicillin	249	Lys143	Thr11,Lys70	Arg84,Thr69	−4.97	−6.91	−8.24	1	2	2
Azithromycin	225	Lys143	NA	Thr69	−5.74	NA	−7.07	1	NA	1
Ceftriaxone	167	Lys143	Thr11,Lys70	Arg84,Thr69	−2.96	−6.2	−7.2	1	2	2
Chloramphenicol	197	Lys143	Thr11,Gly197	Arg84,Thr69	−5.73	−6.85	−6.49	1	2	2
Ciprofloxacin	201	Lys143	Thr12,Lys270	Arg84,Thr69	−5.38	−8.18	−6.92	1	2	2
Digitoxin	236	Lys143	NA	Arg84,Tyr83	−9.89	NA	−8.07	2	NA	2
Ergonovine	240	NA	Thr11,Lys70,Gly197	Arg84,Tyr83	NA	−8.03	−7.63	NA	3	2
Imiquimod	155	NA	Gly197	Arg84,Thr69	NA	−6.69	−7.16	NA	1	2
Isosorbide	172	NA	Thr11,Gly197	Arg84,Thr69	NA	−5.94	−4.84	NA	2	2
Mononitrate										
Levofloxacin	159	Lys143	Thr11,Lys70,Gly197	Arg84,Thr69	−5.49	−7.05	−7.39	1	3	2
Lymecycline	285	NA	Thr11,Lys70	Arg84,Thr69	NA	−8.05	−9.29	NA	2	2
Ofloxacin	174	Lys143	Thr11,Lys70,Gly197	Arg84,Thr69	−5.4	−7.69	−7.53	1	3	2
Rimonabant	104	NA	NA	Arg84	NA	NA	−9.21	NA	NA	1
Rolitetracycline	223	NA	NA	Arg84,Thr69	NA	NA	−9.45	NA	NA	2
Sulfamethoxazole	201	Lys143	Thr11,Lys70,Gly197	Arg84,Thr69	−5.22	−7.22	−8.29	1	3	2
Trimethoprim	263	Lys143	Thr11, Thr12, Lys55	Arg84,Thr69	−4.24	−7.4	−6.55	1	3	2
**XR770**	**197**	**Lys143**	**Thr11,Gly197**	**Arg84,Thr69**	**-9.98**	**-8.76**	**-11.48**	**1**	**2**	**2**
XR587	129	NA	Thr11,Lys70,Gly197	Thr69	NA	−7.04	−7.69	NA	3	1

*Bold values indicates best representative*.

**Table 5:** Interaction pattern of SicA, DnaK, and SigE with the corresponding interacting residues and binding energy against non-typhoidal drugs.

**Drugs**	**SASA**	**Interacting residues**			**Binding energy**			**H bonds**		
	**(A^2^)**	**SicA**	**DnaK**	**SigE**	**SicA**	**DnaK**	**SigE**	**SicA**	**DnaK**	**SigE**
Moxifloxacin	265	Lys143	Lys70	Arg84	−5.21	−6.02	−6.30	1	1	1
Grepafloxacin	187	Lys143,Thr21	Thr11,Gly197	Thr69,Arg84	−6.42	−6.38	−6.31	1	2	2
Lomefloxacin	148	Lys143	Lys70, Thr11	Arg84	−5.96	−6.22	−7.24	1	2	1
Gatifloxacin	214	Lys143,Thr21	Thr11,Gly197	NA	−6.76	−6.98	−6.67	2	2	NA
Sparfloxacin	216	Lys143	Thr11,Lys70	Thr69,Arg84	−5.18	−6.82	−5.23	1	2	2
Temafloxacin	191	Lys143	Gly197	Arg84	−6.11	−6.06	−6.15	1	1	1
Nemonoxacin	230	Thr21	Thr11, Lys70	Thr69	−6.48	−6.32	−6.33	1	2	1
Besifloxacin	228	Lys143	NA	NA	−4.20	NA	NA	1	NA	NA
Finafloxacin	165	NA	Gly197, Lys70	NA	−5.28	NA	NA	NA	2	NA
Cadazolid	214	Lys143,Thr21	Thr11, Gly197	Arg84	−5.94	−6.00	−5.32	2	2	1
Nadifloxacin	148	Lys143	NA	Arg84,Thr69	−7.21	NA	−6.29	1	NA	2
**Sitafloxacin**	**191**	**Lys143,Thr21**	**Thr11,Lys70,Gly197**	**Arg84,Thr69**	**-7.57**	**-7.84**	**-7.39**	**2**	**3**	**2**
Clinafloxacin	264	NA	Thr11, Gly197	NA	NA	−7.66	NA	NA	2	NA
Pilocarpine	165	Lys143	Thr11, Gly197	Arg84	−6.60	−6.85	−6.08	1	2	1
Matairesinol	255	Lys143	Thr11,Gly197	Arg84	−6.51	−6.73	−7.52	1	2	2

*Bold values indicates best representative*.

**Table 6:** Interaction pattern of SicA, DnaK, and SigE with the corresponding interacting residues and Binding energy against Hsp70 modulators.

**Drugs**	**SASA**	**Interacting residues**			**Binding energy**			**H bonds**		
	**(A^2^)**	**SicA**	**DnaK**	**SigE**	**SicA**	**DnaK**	**SigE**	**SicA**	**DnaK**	**SigE**
EGCG	151	Lys143	Gly197,Thr11	Arg84,Thr69	−6.93	−10.54	−10.87	1	2	2
MKT-077	189	NA	Lys121	Arg84	NA	−7.24	−9.07	NA	2	1
SW02	143	NA	Lys70	Arg84,Thr69	NA	−7.95	−8.21	NA	2	1
115–7c	146	Lys143	Gly197,Thr12	Arg84,Thr69	−7.31	−8.69	−9.86	1	2	2
**Compound 1**	**229**	**Lys143**	**Thr12**	**Arg84,Thr69**	−**8.35**	−**8.39**	−**10.51**	**1**	**1**	**1**
NSC-630668	183	NA	NA	Arg84,Thr69	NA	NA	−8.02	NA	NA	NA
MAL3-101	158	Lys143	NA	Arg84,Thr69	−8.69	NA	−6.53	1	NA	NA

*Bold values indicates best representative*.

### Molecular dynamics simulation of SicA, DnaK, and SigE protein complex from *S*. typhimurium and *S*. typhi

To study the macromolecular interactions of SicA, DnaK, and SigE with XR770, simulation studies were performed using MOE (Chemical Computing Group, Montreal). Multiple conformations of the SicA, DnaK, and SigE complex were prepared based on minimum binding energy and number of hydrogen bonds. For the SicA-XR770 protein ligand complex, three docked poses were simulated as three replicates. All structures were corrected using the clean protein application in Discovery Studio (DS) 2.5 (Accelrys Inc., San Diego, CA). This preparation of all molecular files followed by Molecular Dynamics (MD) simulations were conducted using the previously described energy minimized files. The all-atom CHARMM 27 force field was assigned to all molecules for topology generation, and the explicit extended simple point charge (SPC/E) SPC216 water model was applied to solvate the molecules. A triclinic box was generated with a minimum of 1.0 nm distance from the edges of the box to maintain periodic boundary conditions throughout the simulations. Adequate counter ions (Cl^−^ and Na^+^) were added to the solvent to keep the system neutral at physiological ionic strength (0.10 M salt concentration). Steepest descent minimization was performed until the maximum force reached below 1,000.0 kJ mol^−1^ nm^−1^. Before the MD simulations, the systems were equilibrated using position restrained (PR) for 100 ps of isochoric-isothermal (NVT) equilibration at 300K. This was followed by an equilibration under an isothermal-isobaric (NPT) ensemble for 100 ps at the same temperature and 1.0 bar of pressure without position restraints. In NVT and NPT ensembles, the short range non-bonded interactions were defined as van der Waals (VDW) and electrostatic interactions for particles within 1.0 nm. The well-equilibrated systems SicA-XR770, DnaK-XR770, and SigE-XR770 were used to run 100 ns production of MD simulation in three replicates using velocity rescale thermostat. The long-range electrostatic interactions i.e., Particle Mesh Ewald (PME) treatments were also implemented as described in NVT and NPT ensembles (Hansson et al., 2002; Shattuck, 2011).

## Results

### Docking analysis

**Figure 3:**
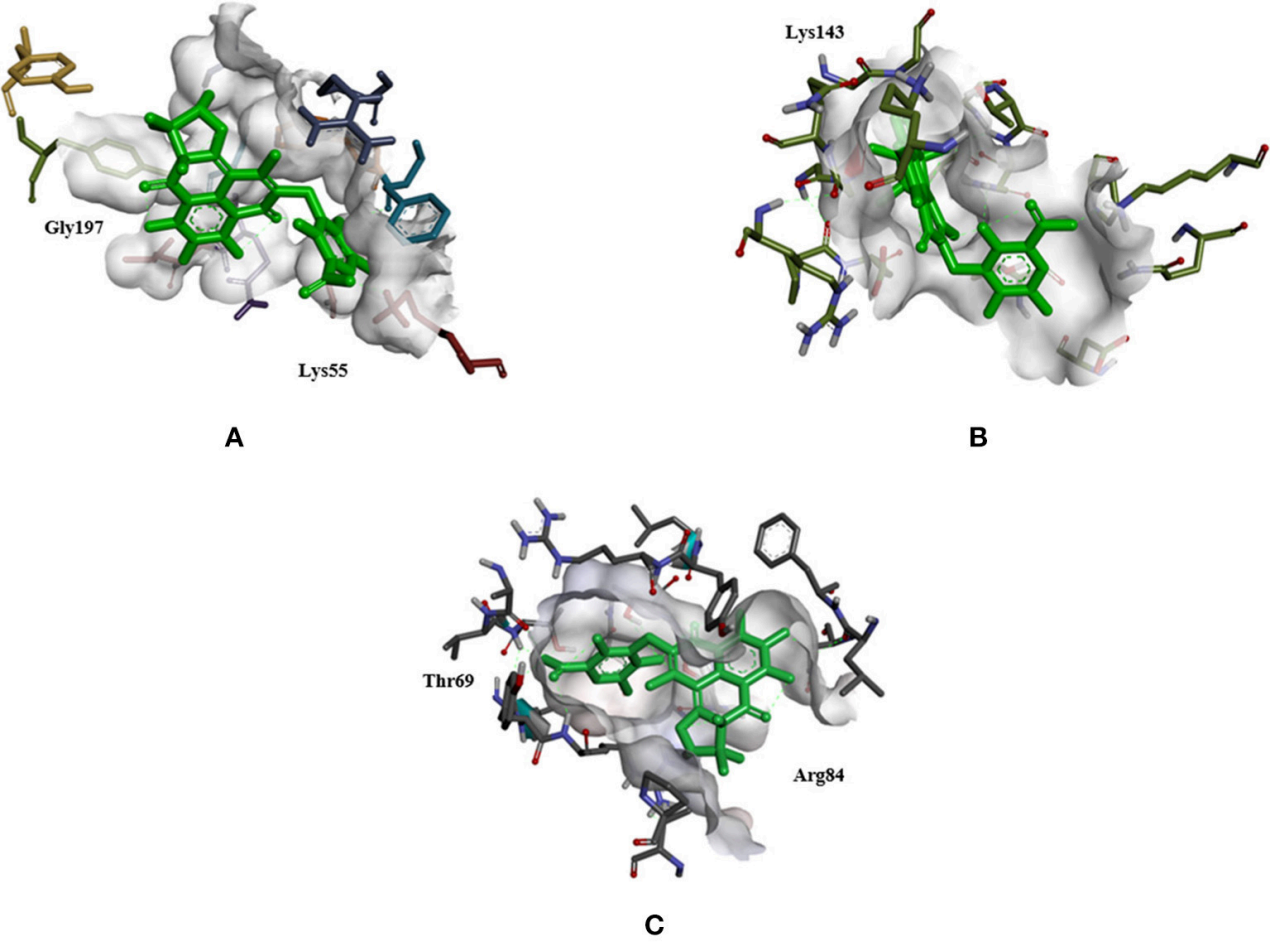
The best docked complex of DnaK, SicA, and SigE with XR770, a histidine kinase inhibitor. **(A)** DnaK with XR770, interacts with −8.76 binding energy, the target residues being Gly197 and Lys55. **(B)** SicA with XR770, interacts with −9.78 binding energy, the target residue being Lys143. **(C)** SigE with XR770, interacts with −11.48 binding energy, the target residues being Thr69 and Arg84.

Docking analysis of all the docked complexes of the aforementioned proteins viz. SicA, Dnak, SigE, SsrA, SsrB, OmpR, and EnvZ against the set of 19 typhoid related drugs, 15 non-typhoidal drugs and 7 Hsp70 modulators was performed. The docking outcome was analyzed on the source of ranked clusters of compound conformations with the binding energy values of various ligands as shown in Tables 4–6. High affinity is related to a high release of the free energy upon binding. The binding pattern of XR770 and XR587, along with the list of marketed drugs against typhoid and diarrhea, were compared for their efficiency against SicA, DnaK, and SigE. Based on the binding pattern of the aforementioned proteins, SicA showed Lys143 as the most consistent residue for all the drugs with the highest binding energy being −9.98 kcal/mol for XR770. For DnaK, the prominent residues were Thr11 and Gly197 showing the highest binding energy (−8.76 kcal/mol) with XR770. Similarly, SigE showed Thr69 and Arg84 to be the residues at the active sites for all the drugs. Interestingly, XR770 showed the highest binding energy of −11.48 kcal/mol with SigE which, notably, was the strongest binding affinity amongst all the proteins compared. Moreover, the chaperone proteins i.e., SicA, DnaK, and SigE, showing commendable interaction with efficient binding energies with XR770, were considered for further molecular dynamics simulation studies (Figure 3).

### Binding interactions between SicA, DnaK, and SigE with XR770 protein-ligand complex

Protein-ligand interaction depends on some bonded and non-bonded interactions. To make a stable protein-ligand complex, as well as for proper folding of proteins several H-bonds, nonbonded electrostatic and van-der Waals (vdW) interactions (Table 7 and Supplementary Figure 5). Among the amino acid residues of SicA, the residues participating in protein-ligand formation are Lys143 for *S*. Typhimurium and Arg61, Gln86, Lys89 for *S*. Typhi which had more than −70 kJ mol^−1^ total electrostatic and vdW energy terms. Similarly, in DnaK Thr11, Gly197 for *S*. Typhimurium and Arg167, Asp38, and Ile418 for *S*. Typhi and in SigE Arg84, Thr69 for *S*. Typhimurium and Ala74 for *S*. Typhi are the participating residues. Thus, the amino acid residues from each of the complex i.e., SicA, DnaK, and SigE were maximally involved in the interactions with XR770 throughout MD simulation processes (Supplementary Figure 4). In order to analyse the binding interactions between each chaperone protein complex accurately, hydrogen bond pairing during all frames of the MD production run were calculated in MOE with bond pair distances within 3.6 Å and angles at 35°. It was found that, several amino acid residues formed hydrogen bonds in the protein-ligand complex. MD simulation study showed the clear indication of each amino acid interaction with the hydrogen bond formation. Maximum occupancies of the following amino acids were considered separately for each complex. The solvent accessible surface area of the SicA, DnaK, and SigE complex became higher after simulation. The sum of solvent accessible surface area of SicA, DnaK, and SigE complex was 424 nm^2^ whereas for the DnaK and SigE complex it was 533 and 460 nm^2^, respectively. The spatial changes denote that the XR770 complex with each of the proteins became more accessible for free binding.

**Table 7:** Representative parametric values for predocked and postdocked conformations with respect to temperature, potential energy, pressure and RMSD.

**Protein 3D structure (predocked)**	**Temperature (300K)**	**Potential energy(Kcal/mol)**	**Pressure (bar)**	**RMSD (A^0^)**
SicA	298	−2942.36	130.0	1.15
DnaK	299	−2761.78	139.2	1.21
SigE	298	−2789.245	125	1.17
**Protein-ligand complex (postdocked)**	**Temperature (300K)**	**Potential energy(Kcal/mol)**	**Pressure (bar)**	**RMSD (A**^0^**)**
SicA-XR770	299	−2301.36	145.5	1.32
DnaK–XR770	296	−2263.78	160.10	1.19
SigE–XR770	298	−2298.245	170	1.26

## Discussion

Multidrug resistance has increased dramatically in the last two decades. Therefore, drug repurposing, is gaining in importance. The analysis and prediction of the activity of existing and novel drug ligands for new protein targets is based on the concept that similar compounds tend to have similar biological properties. Similarly, incorporating a structure based approach focusses on obtaining proteins likely to have similar functions and/or to recognize similar ligands. Thus, in the field of drug repurposing, protein comparison is used as a method to identify secondary targets of an approved drug. To hypothesize a new target for treating MDR *Salmonella*, we have explored the results of a network based approach (Lahiri et al., 2014) to identify the most indispensable proteins important for available as well as next generation drugs. The present study further develops this work at a structural level.

Furthermore, recent progress in proteomics has significantly increased the plausible number of macromolecular targets as candidates for drug discovery. Molecular docking studies have been successfully exploited in drug repurposing. Such techniques are used to predict the geometry and binding energy of the interaction of a protein in complex with a small-molecule ligand. Therefore, the method can be used to predict if a given drug is potentially able to bind other targets. The ability of such target proteins to be bound by the approved drugs shows the druggability of these proteins and indicates their potential as drug targets for the treatment of disease. Additionally, the binding between these proteins and the drugs could also probably indicate their involvement in the mechanism of action of the drugs. Such mechanism might entail inhibition of the drugs, which is exhibited computationally through the efficiency of docking between proteins and ligands, and largely depends upon the binding energy. Through molecular simulation studies, we have observed the stability in the binding energy of the target-ligand complexes and consistency of their interacting residues.

We therefore gain an insight into the ligand specificity of a protein which depends on the consistently binding active site residues of the target proteins. Thus, the homolog*y* models of SicA, DnaK, SigE, SsrA, SsrB, EnvZ, and OmpR were used for further docking studies (Table 1). Among the selected drugs *viz*. 19 typhoid related, 15 non-typhoidal, and 7 Hsp70 modulators, some do not obey Lipinski's rule of 5 but are still in the market and thus, were further considered for drug repositioning (Tables 2, 3). Blind docking was performed for all the chaperones and TCS proteins. This was done to avoid any bias of predictive binding sites of homology modeled proteins and yet, eventually confirm the binding pockets through the same binding residues of each different ligands at the same site, yielding good binding energy against each chaperone and TCS protein. To confirm the binding pocket with the same residues of interaction, loop docking was performed with 35 conformations. To this end, structural alignment of the pockets was also performed for chaperone and TCS proteins against the selected pockets (Supplementary Figure 1). The structural alignment results showed the proper fitting of the pockets as each protein has the same binding site, though the sequence length varies from protein to protein. However, the docking sites of each proteins are different, making it difficult to interpret the difference between them emerging from the structural alignment of the proteins (Supplementary Figure 1).

To have an insight of the same, the most effective conformation, with the highest binding energy for every ligand, was chosen from 30 assigned iterations with the formation of hydrogen bonds between the ligand and protein. Our docking results with XR770 showed hydrogen bond formation with Lys143 of SicA having −9.78 kcal/mol binding energy. Moreover, hydrogen bonds with Lys55 and Gly197 of DnaK (−8.76 kcal/mol) and those with Arg84 and Thr69 of SigE (−11.48 kcal/mol) were observed in *S*. Typhimurium (Figure 3). Similarly, *S*. Typhi showed hydrogen bond interactions with Arg61, Gln86, and Lys89 of SicA, Arg167, Asp388, and Ile418 of DnaK and Ala74 of SigE, with respective binding energies of −9.85, −9.57, and −7.35 kcal/mol (Tables 4, 5). These interacting residues showed consistency and thus, ligand specificity, conferring highest binding energy from the available conformations. Notably, XR770, Sitafloxacin and Compound 1 topped the best in the individual set amongst the three sets of drugs i.e., 19 typhoid related, 15 non-typhoidal, and 7 Hsp70 modulators, respectively. Again, XR587 showed comparatively slightly less binding energy than XR770, which has the probability to show better results if explored against different selected targets. Even though functions of some of the non-typhoidal drugs are unknown (Supplementary Table 2), they show interaction against the selected proteins with lower binding energies compared to XR770. We have also incorporated pharmacophore based prediction to analyse the common features that reveal the activity of the drugs. However, pharmacophore based screening was not performed because our objective was to check the available marketed typhoidal and non-typhoidal drugs against the target proteins selected i.e., chaperone and TCS. The common pharmacophore features for the best binding drugs are shown in Supplementary Figure 6. These observations might help us to delineate the broader perspective of typhoid drug repositioning by either considering XR770 with the fused phenaleno-furanone moiety as the repurposed ligand or the chaperone proteins like SicA, DnaK, and /or SigE as the network based new targets and pose them for experimental studies for revealing potential side-effects, if any.

To determine the efficacy of inhibition, a comparative account of the aforementioned binding patterns of XR770 showed it to be conferring the greatest binding affinity among the selected anti-typhoid drugs, like Amoxicillin and Ciprofloxacin (Table 2). In fact, resistance to the approved drugs, like Amoxicillin and Ciprofloxacin, in systemic infection, has been known in patients with typhoid and diarrhea (Cui et al., 2008). Besides, these drugs are also known to cause many unpleasant side effects, such as stomach pain, nausea, vomiting, vaginal itching or discharge, headache etc. (Leegaard et al., 1996). The toxicity for patients against typhoidal and non-typhoidal drugs is given in Supplementary Tables 3, 4.

Notably, XR770 in comparison to other drugs in this study showed more consistent amino acid residues and highest binding affinity toward the target proteins. Moreover, the greater number of hydrogen bonding formed suggests more stability of the binding interactions as are also conceived through molecular dynamics simulation. Furthermore, in order to address the issue about specificity, the parameters like relatively high difference in binding energy and binding to specific active site residues in the protein has been considered to project XR770 as the potential candidate.

Pertaining to the above results observed, thus, proving the effectiveness of the aforementioned selected proteins as drug targets could be the real challenge. To cater to this need, we have incorporated other known virulent TCS proteins of *Salmonella* to be compared with the chaperones. In all the cases, XR770 turned out to be the promising chemical ligand in inhibiting the selected targets *viz*. SicA, DnaK, SigE, SsrA, SsrB, EnvZ, and OmpR. Based on the least energy score, best docked complex was compared with the interaction pattern of XR770. Comparative study of docking interaction pattern of the above listed proteins was also performed in *Salmonella* typhi as well as shown in Supplementary Table 1 which revealed similar results.

Potency of a drug does not depend solely on its overall binding affinity with the target(s). On the contrary, it is a result of the complex interaction of the drug binding efficacy, namely the ability of the drug to exhibit a biological response upon interaction with the target protein. This interaction may be an agonist or antagonist depending on the physiological response. Based on the docking energy scores and ADME properties, it was found that XR770 has lower energy scores revealing higher binding affinity toward the active site. The binding follows Lipinski's rule of five with a little higher molecular weight which is acceptable when compared to several marketed drugs.

The binding free energies for the stable protein-ligand complexes with XR770 for SicA, DnaK, and SigE were −2301.36, −2263.78, and −2298.245 kJ mol^−1^, which also denote stability with respect to temperature and pressure (Supplementary Figure 5 and Table 7). The results of the MD simulation run clearly indicated that these protein-ligand complexes were held together by strong intermolecular non-covalent forces. These strong binding interactions between the XR770 and chaperone proteins make the complexes highly stable and fit for possible involvement in various reactions (Supplementary Figure 4).

Despite having such exemplary computational binding activities against the chaperone and TCS proteins, it is worthwhile to mention that XR770 has been reported to have only inhibitory effect on NRII (or NtrB) histidine kinase from *E. coli* but no effect is reported for the whole cell bacteria (Chrystal et al., 2007; Bem et al., 2014). However, in the absence of proper knowledge of the assay done to check for the activity of XR770, it can be considered as the main caveat of this study.

## Conclusion

From our study, chaperone proteins SicA, DnaK, and SigE along with the TCS proteins SsrA SsrB, EnvZ, and OmpR have been demonstrated to be potential druggable targets for the new phenaleno-furanone based ligand XR770.Thus, XR770 confers high binding affinity toward all the target proteins with a binding affinity comparable to the approved drugs. Hence, XR770 might prove to be potent inhibitor for the chaperone proteins. However, pharmacological studies are required to confirm the inhibitory activity of XR770 against the chaperones as effective drug targets.

## Author contributions

CL conceived the concepts, planned, and designed the layout of the work. The work was conducted by SM under the primary supervision of CL with additional supervisory input from DG. Figures were created by SM. SM wrote the paper, with textual revisions by CL and DG.

### Conflict of interest statement

The authors declare that the research was conducted in the absence of any commercial or financial relationships that could be construed as a potential conflict of interest.

## References

[B1] Aubé J. (2012). Drug repurposing and the medicinal chemist. ACS Med. Chem. Lett. (2012) 3, 442–444. 10.1021/ml300114c 24900492 PMC4025634

[B2] Bem A. E. Velikova N. Pellicer M. T. Baarlen P. V. Marina A. Wells J. M. (2014). *Bacterial histidine* kinases as novel antibacterial drug targets. ACS Chem. Biol. 10, 213–224. 10.1021/cb5007135 25436989

[B3] Bowie J. U. Luthy R. Eisenberg D. (1991). A method to identify protein sequences that fold into a known three-dimensional structure. Science 253, 164–170. 1853201 10.1126/science.1853201

[B4] Calderwood S. K. Stevenson M. A. Murshid A. (2012). Heat shock proteins, autoimmunity, and cancer treatment. Autoimmune Dis. 2012:486069. 10.1155/2012/486069 23056925 PMC3465951

[B5] Chiappori F. Fumian M. Milanesi L. Merelli I. (2015). DnaK as antibiotic target: hot spot residues analysis for differential inhibition of the bacterial protein in comparison with the human HSP70. PLoS ONE 10:e0124563. 10.1371/journal.pone.0124563 25905464 PMC4408060

[B6] Chrystal E. J. Wrigley S. K. Thomas R. Nicholson N. Hayes M. (eds.). (2007). Biodiversity: New Leads for the Pharmaceutical and Agrochemical Industries. Cambridge, UK: Royal Society of Chemistry.

[B7] Csizmadia P. (1999). MarvinSketch and MarvinView: molecule applets for the World Wide Web, in Proceedings of ECSOC-3, the Third International Electronic Conference on Synthetic Organic Chemistry, September 1a30 (Basel), 367–369.

[B8] Cui S. Li J. Sun Z. Hu C. Jin S. Guo Y.. (2008). Ciprofloxacin-resistant *Salmonella enterica* serotype Typhimurium, China. Emerg. Infect. Dis. 14:493. 10.3201/eid1403.070857 18325271 PMC2570801

[B9] Darwin K. H. Miller V. L. (2001). Type III secretion chaperone-dependent regulation: activation of virulence genes by SicA and InvF in Salmonella typhimurium. EMBO J. 20, 1850–1862. 10.1093/emboj/20.8.1850 11296219 PMC125432

[B10] Darwin K. H. Robinson L. S. Miller V. L. (2001). SigE is a chaperone for the *Salmonella enterica* serovar Typhimurium invasion protein SigD. J. Bacteriol. 183, 1452–1454. 10.1128/JB.183.4.1452-1454.2001 11157959 PMC95020

[B11] Dundas J. Ouyang Z. Tseng J. Binkowski A. Turpaz Y. Liang J. (2006). CASTp: computed atlas of surface topography of proteins with structural and topographical mapping of functionally annotated residues. Nucleic Acids Res. 34(Suppl. 2), W116–W118. 10.1093/nar/gkl282 16844972 PMC1538779

[B12] Fink A. L. (1999). Chaperone-mediated protein folding. Physiol. Rev. 79, 425–449. 10.1152/physrev.1999.79.2.425 10221986

[B13] Friesner R. A. Murphy R. B. Repasky M. P. Frye L. L. Greenwood J. R. Halgren T. A.. (2006). Extra precision glide: Docking and scoring incorporating a model of hydrophobic enclosure for protein– ligand complexes. J. Med. Chem. 49, 6177–6196. 10.1021/jm051256o 17034125

[B14] Hansson T. Oostenbrink C. van Gunsteren W. (2002). Molecular dynamics simulations. Curr. Opin. Struct. Biol. 12, 190–196. 10.1016/S0959-440X(02)00308-1 11959496

[B15] Kaur S. P. Rao R. Nanda S. (2011). Amoxicillin: a broad spectrum antibiotic. Int. J. Pharm. Pharm. Sci. 3, 30–37.

[B16] Kelley L. A. Mezulis S. Yates C. M. Wass M. N. Sternberg M. J. (2015). The Phyre2 web portal for protein modeling, prediction and analysis. Nat. Protoc. 10:845. 10.1038/nprot.2015.053 25950237 PMC5298202

[B17] Kitchen D. B. Decornez H. Furr J. R. Bajorath J. (2004). Docking and scoring in virtual screening for drug discovery: methods and applications. Nat. Rev. Drug Discov. 3:935. 10.1038/nrd1549 15520816

[B18] Lahiri C. Pawar S. Sabarinathan R. Ashraf M. I. Chand Y. Chakravortty D. (2014). Interactome analyses of Salmonella pathogenicity islands reveal SicA indispensable for virulence. J. Theor. Biol. 363, 188–197. 10.1016/j.jtbi.2014.08.013 25128737

[B19] Leegaard T. M. Van Gestel M. H. Petit P. L. C. Van De Klundert J. A. M. (1996). Antibiotic resistance mechanisms in Salmonella species causing bacteraemia in Malawi and Kenya. Apmis 104, 302–306. 10.1111/j.1699-0463.1996.tb00721.x 8645470

[B20] Liberek K. Lewandowska A. Zietkiewicz S. (2008). Chaperones in control of protein disaggregation. EMBO J. 27, 328–335. 10.1038/sj.emboj.7601970 18216875 PMC2234349

[B21] March-Vila E. Pinzi L. Sturm N. Tinivella A. Engkvist O. Chen H.. (2017). On the integration of *in silico* drug design methods for drug repurposing. Front. Pharmacol. 8:298. 10.3389/fphar.2017.00298 28588497 PMC5440551

[B22] Morris G. M. Huey R. Lindstrom W. Sanner M. F. Belew R. K. Goodsell D. S.. (2009). AutoDock4 and AutoDockTools4: automated docking with selective receptor flexibility. J. Comput. Chem. 30, 2785–2791. 10.1002/jcc.21256 19399780 PMC2760638

[B23] Neckers L. Tatu U. (2008). Molecular chaperones in pathogen virulence: emerging new targets for therapy. Cell Host Microbe 4, 519–527. 10.1016/j.chom.2008.10.011 19064253 PMC2752846

[B24] Pan A. Lahiri C. Rajendiran A. Shanmugham B. (2015). Computational analysis of protein interaction networks for infectious diseases. Brief. Bioinformatics 17, 517–526. 10.1093/bib/bbv059 26261187 PMC7110031

[B25] Patury S. Miyata Y. Gestwicki J. E. (2009). Pharmacological targeting of the Hsp70 chaperone. Curr. Top. Med. Chem. 9, 1337–1351. 10.2174/156802609789895674 19860737 PMC2799686

[B26] Rangel-Vega A. Bernstein L. R. Mandujano Tinoco E. A. García-Contreras S. J. García-Contreras R. (2015). Drug repurposing as an alternative for the treatment of recalcitrant bacterial infections. Front. Microbiol. 6:282. 10.3389/fmicb.2015.00282 25914685 PMC4391038

[B27] Schwede T. Kopp J. Guex N. Peitsch M. C. (2003). SWISS-MODEL: an automated protein homology-modeling server. Nucleic Acids Res. 31, 3381–3385. 10.1093/nar/gkg520 12824332 PMC168927

[B28] Shattuck T. W. (2011). Colby College Molecular Mechanics Exercises MOE (Molecular Operating Environment) Exercises. Montreal, QC: Chemical Computing Group ULC.

[B29] Sivakumar D. Lahiri C. Chakravortty D. (2013). Computational studies on histidine kinase protein BaeS to target multidrug-resistant Salmonella. Med. Chem. Res. 22, 1804–1811. 10.1007/s00044-012-0188-6

[B30] Sousa S. F. Fernandes P. A. Ramos M. J. (2006). Protein–ligand docking: current status and future challenges. Proteins Struct. Funct. Bioinformatics 65, 15–26. 10.1002/prot.21082 16862531

[B31] Trott O. Olson A. J. (2010). AutoDock Vina: improving the speed and accuracy of docking with a new scoring function, efficient optimization, and multithreading. J. Comput. Chem. 31, 455–461. 10.1002/jcc.21334 19499576 PMC3041641

[B32] Tucker S. C. Galán J. E. (2000). Complex function for SicA, a *Salmonella enterica* serovar typhimurium type III secretion-associated chaperone. J. Bacteriol. 182, 2262–2268. 10.1128/JB.182.8.2262-2268.2000 10735870 PMC111276

[B33] Wishart D. S. Feunang Y. D. Guo A. C. Lo E. J. Marcu A. Grant J. R.. (2017). DrugBank 5.0: a major update to the DrugBank database for 2018. Nucleic Acids Res. 46, D1074–D1082. 10.1093/nar/gkx1037 29126136 PMC5753335

[B34] Zaidi M. B. Calva J. J. Estrada-Garcia M. T. Leon V. Vazquez G. Figueroa G. (2008). Integrated food chain surveillance system for Salmonella spp. in Mexico. Emerg. Infect. Dis. 14:429 10.3201/eid1403.071057 18325258 PMC2570816

[B35] Zhang Y. (2008). I-TASSER server for protein 3D structure prediction. BMC Bioinformatics 9:40. 10.1186/1471-2105-9-40 18215316 PMC2245901

[B36] Zügel U. Kaufmann S. H. (1999). Role of heat shock proteins in protection from and pathogenesis of infectious diseases. Clin. Microbiol. Rev. 12, 19–39. 9880473 10.1128/cmr.12.1.19 PMC88905

