# Supplementary material for: Paradigm Shift in Drug Re-purposing From Phenalenone to Phenaleno-Furanone to Combat Multi-Drug Resistant Salmonella enterica Serovar Typhi
Source: Front Cell Infect Microbiol. 2018 Nov 14;8:402. doi: 10.3389/fcimb.2018.00402 (PMC6246918; doi:10.3389/fcimb.2018.00402)
Supplement: Supplementary file 1 [file Table_1.docx]

**S. Table 1. Comparison of docking interaction of *S.* Typhimurium and *S*. Typhi proteins with XR770**

| **Proteins** | **Interacting Residues** | | **Binding Energy** | | **Type** |
| --- | --- | --- | --- | --- | --- |
|  | ***S*. Typhimurium** | ***S*. Typhi** | ***S*. Typhimurium** | ***S*. Typhi** |  |
| SicA | Lys143 | Arg61, Gln86,  Lys89 | -9.98 | -9.85 | Chaperone |
| DnaK | Thr11,Gly197 | Arg167, Asp38,  Ile418 | -8.76 | -9.57 | Chaperone  (Hsp70s) |
| SigE | Arg84,Thr69 | Ala74 | -11.48 | -7.35 | Chaperone |
| SsrA | His90, Arg133 | Asn450, Lys52,  Ile578 | -8.28 | -10.18 | TCS-RR |
| SsrB | His176 | Arg206,Asn146 | -8.16 | -8.61 | TCS-HK |
| EnvZ | Gly405 | Arg234,Gln292 | -9.56 | -9.25 | TCS-HK |
| OmpR | Ser163,Arg207 | Arg71 | -9.15 | -9.69 | TCS-HK |
|  |  |  |  |  |  |
