# Supplementary material for: Paradigm Shift in Drug Re-purposing From Phenalenone to Phenaleno-Furanone to Combat Multi-Drug Resistant Salmonella enterica Serovar Typhi
Source: Front Cell Infect Microbiol. 2018 Nov 14;8:402. doi: 10.3389/fcimb.2018.00402 (PMC6246918; doi:10.3389/fcimb.2018.00402)
Supplement: Supplementary file 3 [file Table_3.docx]

**S. Table 3. Relative toxicity for the patients against Typhoidal drugs**

| **Drug** | **Toxicity** |
| --- | --- |
| Acenocoumarol | The onset and severity of the symptoms are dependent on the individual's sensitivity to oral anticoagulants, the severity of the overdosage, and the duration of treatment. Bleeding is the major sign of toxicity with oral anticoagulant drugs. The most frequent symptoms observed are: cutaneous bleeding (80%), haematuria (with renal colic) (52%), haematomas, gastrointestinal bleeding, haematemesis, uterine bleeding, epistaxis, gingival bleeding and bleeding into the joints. Further symptoms include tachycardia, hypotension, peripheral circulatory disorders due to loss of blood, nausea, vomiting, diarrhoea and abdominal pains. |
| Alverine | Can produce hypotension and atropine-like toxic effects. Fatality has occurred following overdose with very high doses. |
| Amoxicillin | Serious toxicity is unlikely following large doses of amoxicillin. Acute ingestion of large doses of amoxicillin may cause nausea, vomiting, diarrhea and abdominal pain. Acute oliguric renal failure and hematuria may occur following large doses. |
| Anacardic Acid | NA |
| Auranofin | Oral, rat: LD_50_ = > 2000 mg/kg. Symptoms of overdose may include diarrhoea, vomiting, abdominal cramps, and symptoms of hypersensitivity (such as skin rash, hives, itching, and difficulty breathing). |
| Azithromycin | Potentially serious side effects of angioedema and cholestatic jaundice were reported |
| Ceftriaxone | NA |
| Chloramphenicol | Oral, mouse: LD_50_ = 1500 mg/kg; Oral, rat: LD_50_ = 2500 mg/kg. Toxic reactions including fatalities have occurred in the premature and newborn; the signs and symptoms associated with these reactions have been referred to as the gray syndrome. Symptoms include (in order of appearance) abdominal distension with or without emesis, progressive pallid cyanosis, vasomotor collapse frequently accompanied by irregular respiration, and death within a few hours of onset of these symptoms. |
| Ulexone C | NA |
| CID_21591963 | NA |
| Ulexin C | NA |
| Osajin | NA |
| Ciprofloxacin | The major adverse effect seen with use of is gastrointestinal irritation, common with many antibiotics. |
| Digitoxin | Digitoxin exhibits similar toxic effects to the more-commonly used digoxin, namely: anorexia, nausea, vomiting, diarrhoea, confusion, visual disturbances, and cardiac arrhythmias. |
| Doxycycline | Symptoms of overdose include anorexia, nausea, diarrhoea, glossitis, dysphagia, enterocolitis and inflammatory lesions (with monilial overgrowth) in the anogenital region, skin reactions such as maculopapular and erythematous rashes, exfoliative dermatitis, photosensitivity, hypersensitivity reactions such as urticaria, angioneurotic oedema, anaphylaxis, anaphyl-actoid purpura, pericarditis, and exacerbation of systemic lupus erythematosus, benign intracranial hypertension in adults disappearing on discontinuation of the medicine, haematologic abnormalities such as haemolytic anaemia, thrombocytopenia, neutropenia, and eosinophilia. LD_50_=262 mg/kg (I.P. in rat). |
| Ergonovine | The principal symptoms of overdose are convulsions and gangrene. Other symptoms include bradycardia, confusion, diarrhoea, dizziness, dyspnoea, drowsiness, fast and/or weak pulse, miosis, hypercoagulability, loss of consciousness, nausea and vomiting, numbness and coldness of the extremities, pain in the chest, peripheral vasoconstriction, respiratory depression, rise or fall in blood pressure, severe cramping of the uterus, tachycardia, tingling, and unusual thirst. |
| Imiquimod | Symptoms of overdose include flu-like symptoms, such as fever, fatigue, headache, nausea, diarrhoea and muscle pain. |
| Isosorbide_Mononitrate | Symptoms of overdose include vasodilatation, venous pooling, reduced cardiac output, and hypotension. There are no data suggesting what dose of isosorbide mononitrate is likely to be life-threatening in humans. In rats and mice, there is significant lethality at doses of 2000 mg/kg and 3000 mg/kg, respectively. |
| Levofloxacin | Side effects include disorientation, dizziness, drowsiness, hot and cold flashes, nausea, slurring of speech, swelling and numbness in the face |
| Lymecycline | Adverse effects include nausea, vomiting, diarrhoea, glossitis, enterocolitis, dysphagia, dermatitis, hypersensitivity reactions, proctitis, and vaginitis. |
| Ofloxacin | LD_50_=5450 mg/kg (orally in mice) |
| Rimonabant | Almost twice as many people discontinued rimonabant compared with placebo because of adverse events (13.8% vs. 7.2%). These consistently involved psychiatric disorders (8.5% vs. 3.2%), including depression and anxiety. Other common side effects included insomnia, nausea, vomiting, diarrhoea and fatigue. |
| Rolitetracycline | Symptoms of overdose include anorexia, nausea, diarrhoea, glossitis, dysphagia, enterocolitis and inflammatory lesions (with monilial overgrowth) in the anogenital region, skin reactions such as maculopapular and erythematous rashes, exfoliative dermatitis, photosensitivity, hypersensitivity reactions such as urticaria, angioneurotic oedema, anaphylaxis, anaphyl-actoid purpura, pericarditis, and exacerbation of systemic lupus erythematosus, benign intracranial hypertension in adults disappearing on discontinuation of the medicine, haematologic abnormalities such as haemolytic anaemia, thrombocytopenia, neutropenia, and eosinophilia. LD_50_=262 mg/kg (I.P. in rat). |
| Sulfamethoxazole | Sulfamethoxazole may cause nausea, vomiting, diarrhea and hypersensitivity reactions. Hematologic effects such as anemia, agranulocytosis, thrombocytopenia and hemolytic anemia in patients with glucose-6-phosphate dehydrogenase deficiency may also occur. Sulfamethoxazole may displace bilirubin from albumin binding sites causing jaundice or kernicterus in newborns. |
| Trimethoprim | LD_50_=4850 (orally in mice) |
| XR587 | NA |
| XR770 | NA |
| Trimethoprim | LD_50_=4850 (orally in mice) |
| XR587 | NA |
| XR770 | NA |

**Note: NA= Not available**
