# Supplementary material for: Paradigm Shift in Drug Re-purposing From Phenalenone to Phenaleno-Furanone to Combat Multi-Drug Resistant Salmonella enterica Serovar Typhi
Source: Front Cell Infect Microbiol. 2018 Nov 14;8:402. doi: 10.3389/fcimb.2018.00402 (PMC6246918; doi:10.3389/fcimb.2018.00402)
Supplement: Supplementary file 4 [file Table_4.docx]

| **Drugs** | **Toxicity** |
| --- | --- |
| Moxifloxacin | Symptoms of overdose include CNS and gastrointestinal effects such as decreased activity, somnolence, tremor, convulsions, vomiting, and diarrhea. The minimal lethal intravenous dose in mice and rats is 100 mg/kg. |
| Grepafloxacin | Withdrawn from the US market in 1999 due to associations with QTc prolongation and adverse cardiovascular events. |
| Lomefloxacin | Adverse reactions include peripheral neuropathy, nervousness, agitation, anxiety, and phototoxic events (rash, itching, burning) due to sunlight exposure. |
| Gatifloxacin | NA |
| Sparfloxacin | Single doses of sparfloxacin were relatively non-toxic via the oral route of administration in mice, rats, and dogs. No deaths occurred within a 14-day post-treatment observation period at the highest oral doses tested, up to 5000 mg/kg in either rodent species, or up to 600 mg/kg in the dog. Clinical signs observed included inactivity in mice and dogs, diarrhea in both rodent species, and vomiting, salivation, and tremors in dogs. |
| Temafloxacin | Severe adverse reactions, including allergic reactions and hemolytic anemia, developed in about fifty patients during the first four months of its use, leading to three patient deaths |
| Nemonoxacin | NA |
| Besifloxacin | LD50, rat: >2000 mg/kg. The most common adverse reaction reported in 2% of patients treated with besifloxacin was conjunctival redness. |
| Finafloxacin | Finafloxacin was shown to be genotoxic and clastogenic *in vitro*, with and without metabolic activation, and *in vivo*. -General toxicity studies in rats have confirmed sperm toxicity following oral and intravenous dosing. -At 500 mg/kg/day, males were completely infertile, presumably due to low sperm count and sperm immobility. |
| Nadifloxacin | NA |
| Sitafloxacin | NA |
| Clinafloxacin | NA |
| Pilocarpine* | NA |
| Matairesinol* | NA |

**S. Table 4. Relative toxicity for the patients against Non-typhoidal drugs**

**Note: NA = Not available**
