# Supplementary figures and images for: Paradigm Shift in Drug Re-purposing From Phenalenone to Phenaleno-Furanone to Combat Multi-Drug Resistant Salmonella enterica Serovar Typhi
Source: Front Cell Infect Microbiol. 2018 Nov 14;8:402. doi: 10.3389/fcimb.2018.00402 (PMC6246918; doi:10.3389/fcimb.2018.00402)

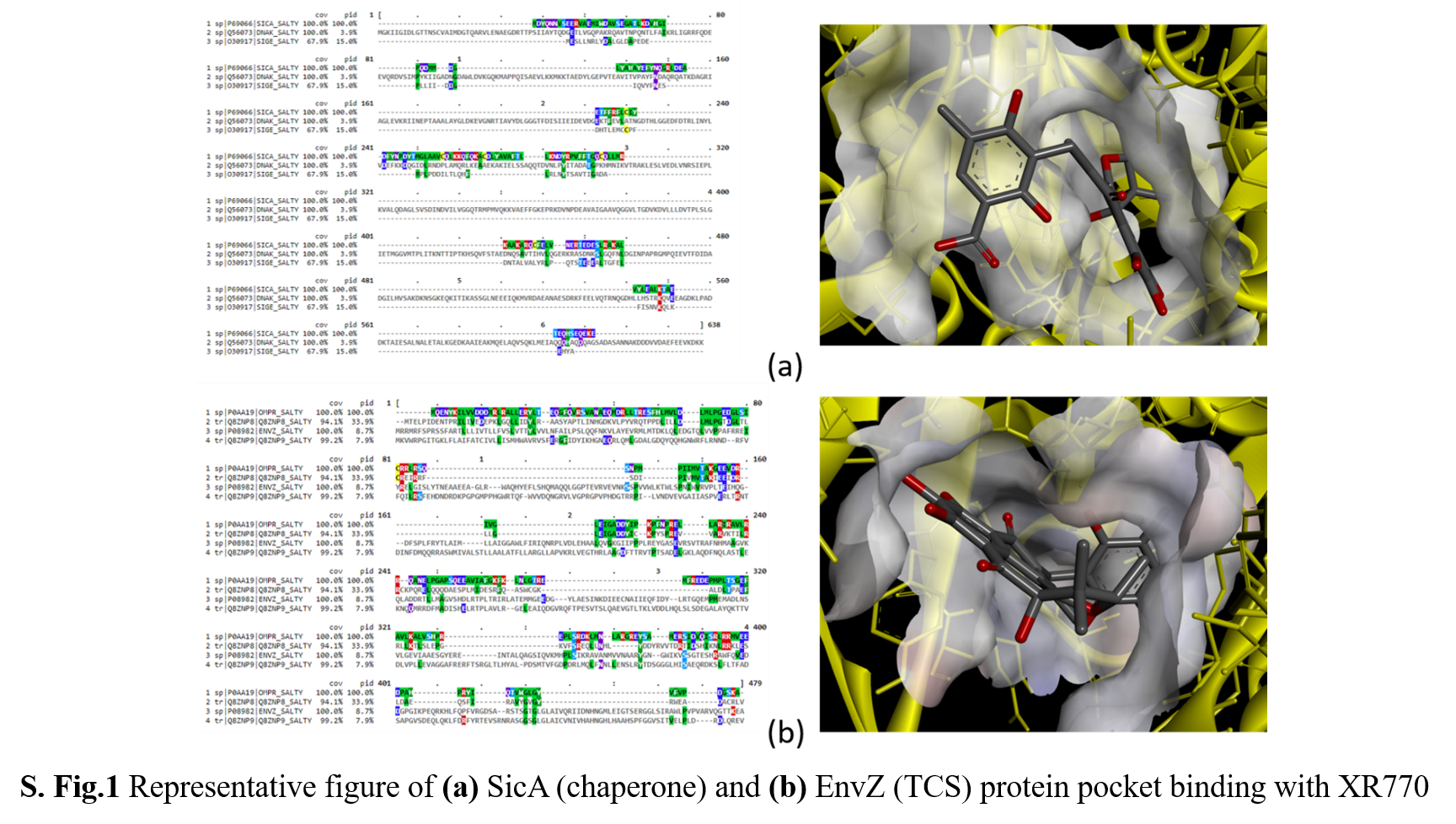

Supplement: Supplementary file 5 [file Image_1.TIF]

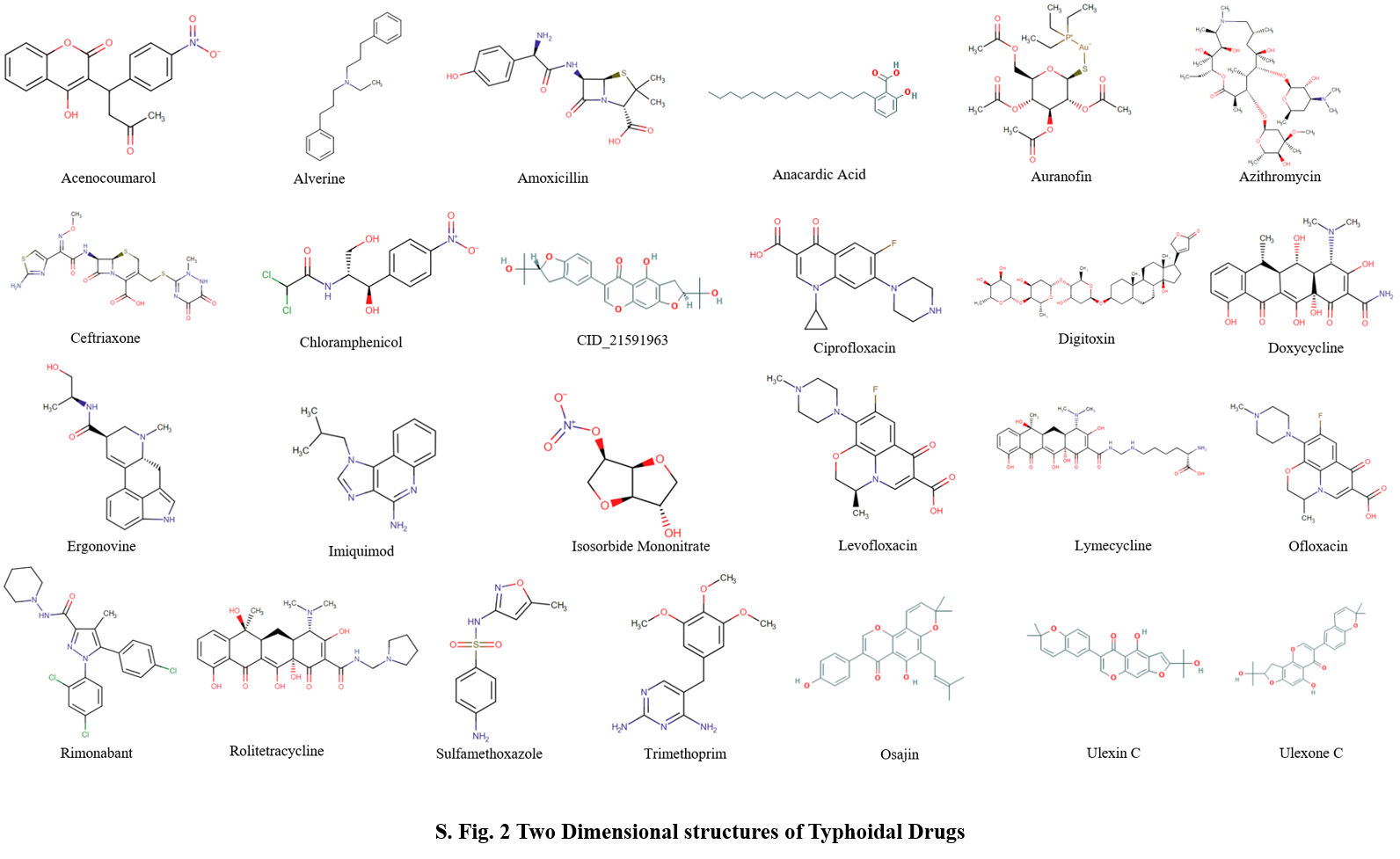

Supplement: Supplementary file 6 [file Image_2.TIF]

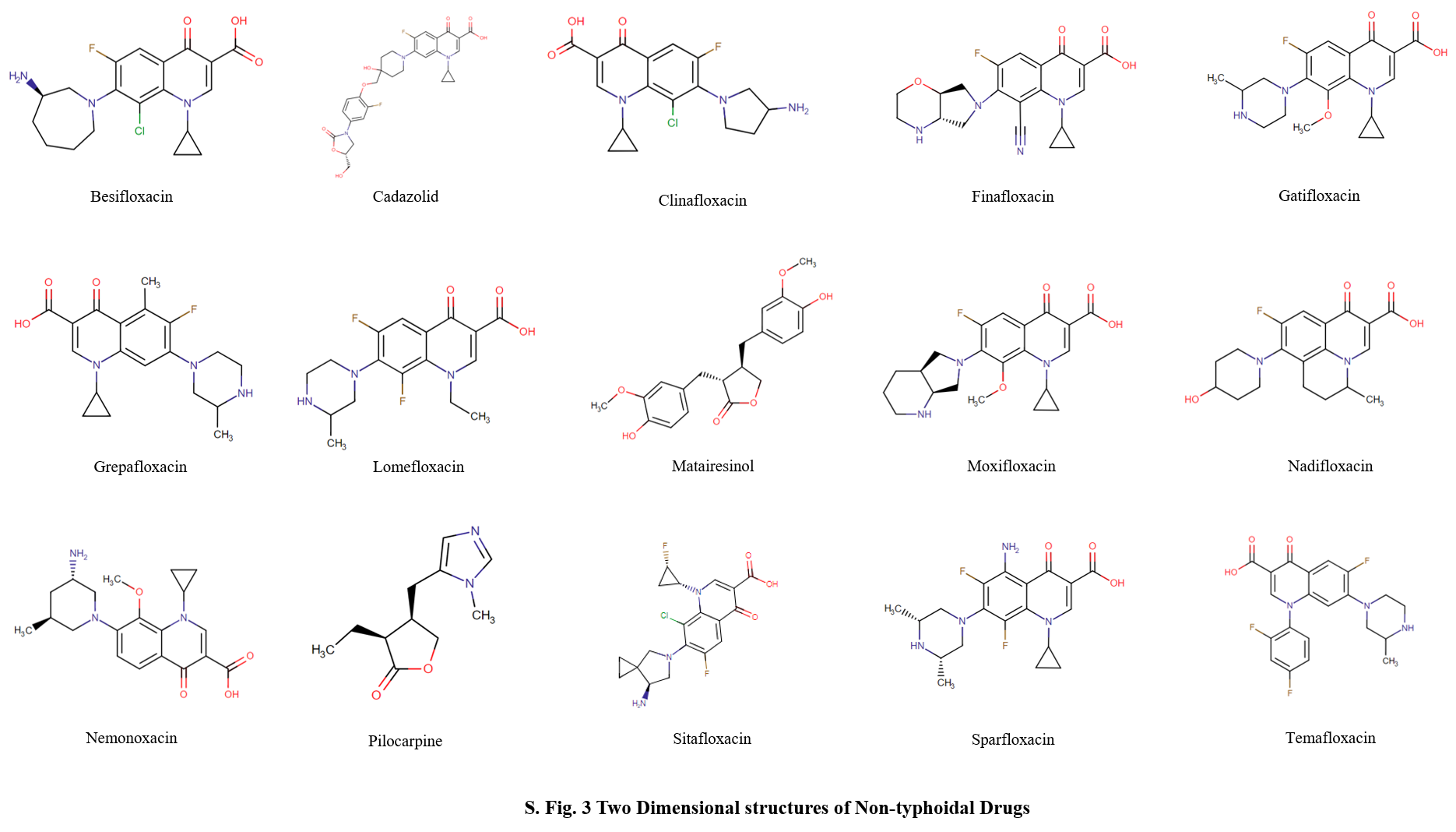

Supplement: Supplementary file 7 [file Image_3.TIF]

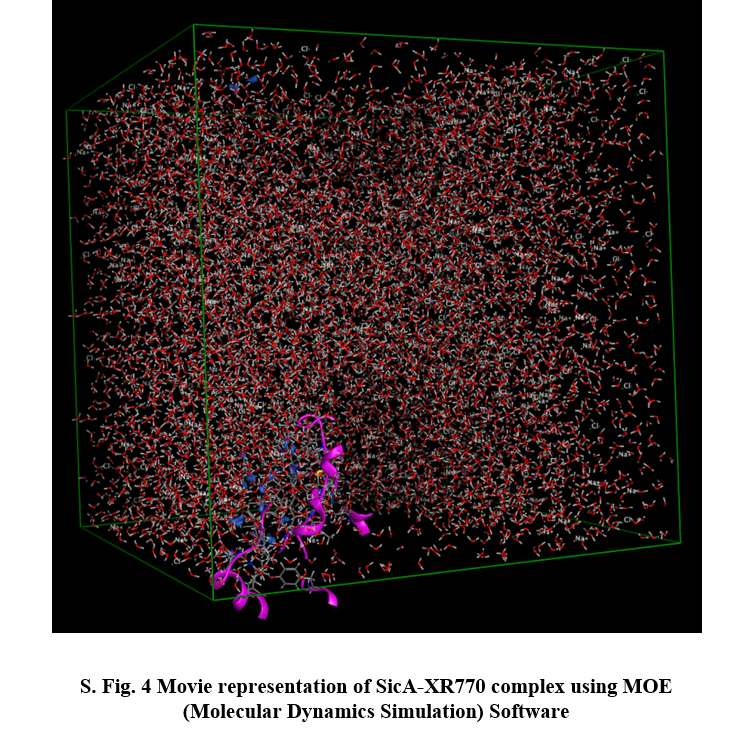

Supplement: Supplementary file 8 [file Image_4.TIF]

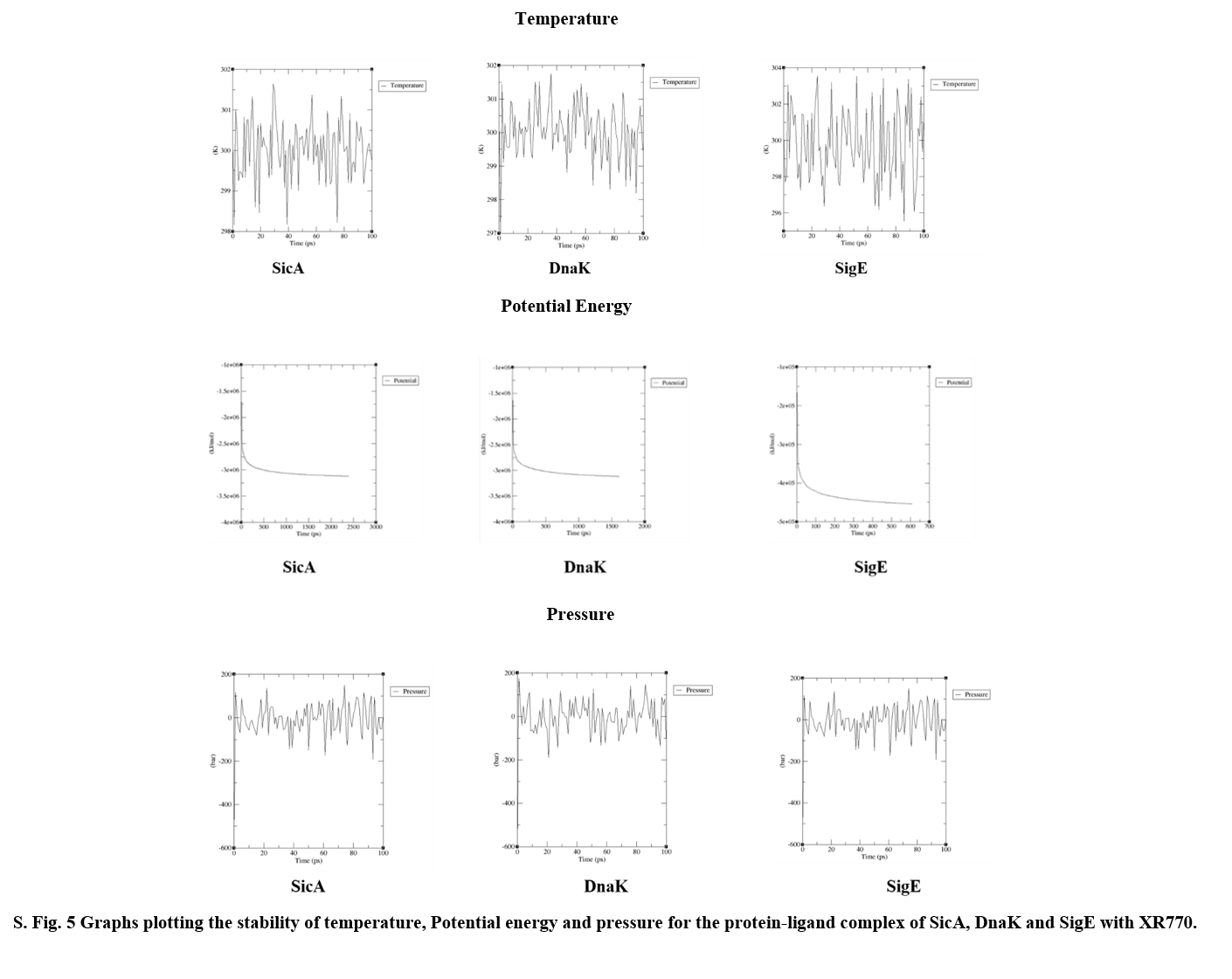

Supplement: Supplementary file 9 [file Image_5.TIF]

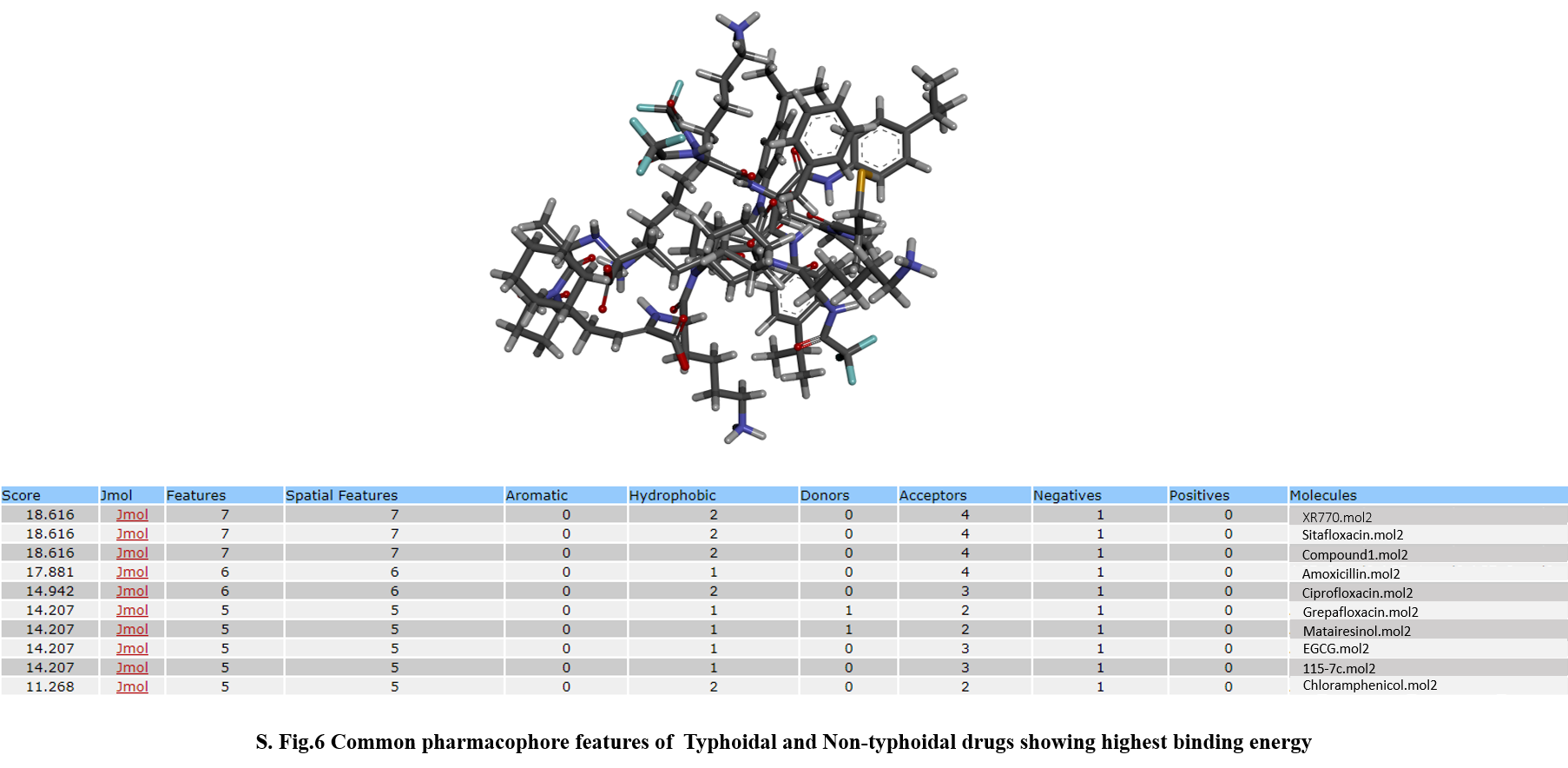

Supplement: Supplementary file 10 [file Image_6.TIF]
